# Natural Bioactive Compounds Targeting Histone Deacetylases in Human Cancers: Recent Updates

**DOI:** 10.3390/molecules27082568

**Published:** 2022-04-15

**Authors:** Abdelhakim Bouyahya, Naoufal El Hachlafi, Tarik Aanniz, Ilhame Bourais, Hamza Mechchate, Taoufiq Benali, Mohammad Ali Shariati, Pavel Burkov, José M. Lorenzo, Polrat Wilairatana, Mohammad S. Mubarak, Nasreddine El Omari

**Affiliations:** 1 Laboratory of Human Pathologies Biology, Department of Biology, Faculty of Sciences, Mohammed V University in Rabat, Rabat 10106, Morocco; ilhame_b@yahoo.com; 2 Microbial Biotechnology and Bioactive Molecules Laboratory, Sciences and Technologies Faculty, Sidi Mohmed Ben Abdellah University, Imouzzer Road Fez, Fez B.P. 1796, Morocco; naoufal.elhachlafi@usmba.ac.ma; 3 Medical Biotechnology Laboratory, Rabat Medical & Pharmacy School, Mohammed V University in Rabat, Rabat B.P. 6203, Morocco; tarik.aanniz@gmail.com; 4 Laboratory of Biotechnology, Environment, Agri-Food and Health (LBEAS), Faculty of Sciences, Sidi Mohamed Ben Abdellah University, Fez B.P. 1796, Morocco; hamza.mechchate@usmba.ac.ma; 5 Environment and Health Team, Polydisciplinary Faculty of Safi, Cadi Ayyad University, Sidi Bouzid B.P. 4162, Morocco; benali.taoufiq@gmail.com; 6 Department of Scientific Research, K. G. Razumovsky Moscow State University of Technologies and Management (The First Cossack University), 73 Zemlyanoy Val, 109004 Moscow, Russia; shariatymohammadali@gmail.com; 7 Center for Biotechnology of Animal Reproduction, South Ural State Agrarian University, 13 Gagarin St., 457100 Troitsk, Russia; burcovpavel@mail.ru; 8 Centro Tecnológico de la Carne de Galicia, Rúa Galicia No.4, Parque Tecnológico de Galicia, 32900 San Cibrao das Viñas, Spain; jmlorenzo@ceteca.net; 9 Área de Tecnoloxía dos Alimentos, Facultade de Ciencias, Universidade de Vigo, 32004 Ourense, Spain; 10 Department of Clinical Tropical Medicine, Faculty of Tropical Medicine, Mahidol University, Bangkok 10400, Thailand; 11 Department of Chemistry, The University of Jordan, Amman 11942, Jordan; 12 Laboratory of Histology, Embryology, and Cytogenetic, Faculty of Medicine and Pharmacy, Mohammed V University in Rabat, Rabat 10100, Morocco; nasrelomari@gmail.com

**Keywords:** epigenetic, histone deacetylases, cancer, natural compounds, epidrugs

## Abstract

Cancer is a complex pathology that causes a large number of deaths worldwide. Several risk factors are involved in tumor transformation, including epigenetic factors. These factors are a set of changes that do not affect the DNA sequence, while modifying the gene’s expression. Histone modification is an essential mark in maintaining cellular memory and, therefore, loss of this mark can lead to tumor transformation. As these epigenetic changes are reversible, the use of molecules that can restore the functions of the enzymes responsible for the changes is therapeutically necessary. Natural molecules, mainly those isolated from medicinal plants, have demonstrated significant inhibitory properties against enzymes related to histone modifications, particularly histone deacetylases (HDACs). Flavonoids, terpenoids, phenolic acids, and alkaloids exert significant inhibitory effects against HDAC and exhibit promising epi-drug properties. This suggests that epi-drugs against HDAC could prevent and treat various human cancers. Accordingly, the present study aimed to evaluate the pharmacodynamic action of different natural compounds extracted from medicinal plants against the enzymatic activity of HDAC.

## 1. Introduction

Cancer is a group of pathologies with severe consequences for human health. It is induced by numerous risk factors, such as genetic predisposition, hormonal disorders, oxidative stress, epigenetic instability, microbial infections, and many others [1]. Recently, close correlations between loss of cellular memory, epigenetic instability, and the onset of certain cancers have been demonstrated [2]. In this respect, epigenetic modifications are biochemical marks deposited on DNA and histones, and they participate in the regulation of gene expression at the transcriptional level. These modifications have several roles, including maintaining cell memory during mitotic division; differentiated cells must retain their cellular memory to keep their identity. However, cells can lose their memory and become cancerous due to epigenetic instability. HDACs play a key role in maintaining repressive gene activity. The repressive activity of HDAC leads to the ectopic repression of various genes, which can affect cellular memory. In addition, normal cells can become cancerous after transformation.

The United States Food and Drug Administration (FDA) has approved four HDAC inhibitors as anti-cancer drugs: Vorinostat, Romidepsin, Belinostat, and Panobinostat. In this respect, Vorinostat (Zolinza^®^) was the first histone HDAC inhibitor approved by the FDA in 2006 to treat advanced and refractory cutaneous T-cell lymphoma (CTCL) [3]. Vorinostat binds to the active sites of histone deacetylases and acts as a chelator of zinc ions at the bottom of the HDAC’s catalytic domain. It causes chromatin decondensation by inhibiting the suppression of acetyl moieties from the ε-amino groups of the lysine residues of histones and transcription factors [4,5]. It, additionally, has a 30% response rate, as revealed by clinical studies [6]. In November 2009, Romidepsin (Istodax^®^), a second HDAC inhibitor, was approved by the FDA for CTCL, with an overall response rate of 34%, and it was later approved for peripheral T-cell lymphoma (PTCL) [6]. According to published research, Vorinostat inhibits the deacetylation of key autophagic markers and interferes with autophagic cell death, which can be an associated mechanism, depending on the involvement of apoptosis [7]. In July 2014, the FDA approved Belinostat (Beleodaq^®^) as a third HDAC inhibitor, to treat patients with relapsed or refractory PTCL, and which has an overall response rate of 25.8% [8]. Another HDAC inhibitor is Panobinostat (Farydak^®^), which was approved in February 2015, to treat multiple myeloma [9]. Furthermore, other HDAC inhibitors, Valproic acid and Entinostat, are in phase III of a clinical trial for use against solid tumors and hormone-receptor-positive advanced breast cancer [10].

Recent investigations revealed that inhibition of HDAC-like enzymes could prevent and treat different types of cancer at the molecular level, by restoring cellular memory. Within this context, specific secondary metabolites extracted from herbs, including terpenoids, phenolic acids, flavonoids, and alkaloids, have shown inhibitory effects against HDAC [11,12,13,14]. These compounds can act specifically, either against the action of HDAC, or against the signaling pathways induced by HDAC [15,16]. Based on the preceding discussion, this review focuses on the close relationship between epigenetic modifications, particularly HDAC enzymes, and tumor transformation. Thus, the pharmacodynamic action of different natural molecules extracted from medicinal plants against the enzymatic activity of HDAC is discussed, to suggest an innovative therapy implementing the maintenance of cellular memory using anti-HDAC epi-drugs.

## 2. Epigenetic Regulation and Cancer

The involvement of environmental, lifestyle, and hereditary factors is the origin of the complexity of cancer etiology. Cancer is most commonly considered a genetic pathology induced by gene expression alteration, following repetitive genetic aberrations. Currently, it is known that the disruption of gene expression causing cell transformation is controlled by epigenetics. Thus, the abnormality of epigenetic regulation has become a model to explain carcinogenesis and cancer development. Research findings suggest that genetic and epigenetic mechanisms are not separate events in cancer, but interact and benefit from each other during tumorigenesis [17,18]. Epigenetic changes are labeled as ‘first hits’ for tumorigeneses. They are the early events responsible for the loss of tissular homeostasis and induce genetic instability; thus, causing changes in the expression profile of tumor-suppressor genes. Furthermore, it is evident that several tumor suppressor genes are rarely genetically mutated but epigenetically silenced [19].

Epigenetic events include histone modifications (phosphorylation, acetylation, methylation, SUMOylation, and ubiquitylation), DNA methylation, and deregulation of non-coding RNAs and their interactions with proteins or nucleic acids [20]. The dynamic regulation of histone marks, DNA, and chromatin structure is dynamically performed by four types of epigenetic regulators, including (i) writers, (ii) erasers, (iii) readers, and (iv) remodelers [21]. Deregulation and mutations in the genes encoding these epigenetic regulators have been described in various cancers [22].

### 2.1. Cancer and DNA Methylation

DNA methylation consisting of a covalent transfer is the most commonly studied epigenetic modification by DNA methyltransferases (DNMTs) of methyl groups at the fifth carbon of cytosine (5-mC) within 5′-CpG-3′ di-nucleotides [23,24]. In mammals, DNMT1, DNMT3a, and DNMT3b are the three main classes of DNMT enzymes. In this regard, abnormal DNA methylation patterns are due to DNMT overexpression or aberrant recruitment. Cancer cells exhibit abnormal DNA methylation patterns, marked by global hypomethylation associated with the promoter and focal hypermethylation of specific genes [25,26]. In addition, aberrant hypomethylation induces the expression of numerous genes, including oncogenes [27], whereas hypermethylation inhibits specific tumor suppressor genes (TSGs) [28]. On the other hand, hypomethylation of oncogenesis is often revealed in cancers such as SLC34A2 in papillary thyroid carcinoma, LY6K in glioblastoma, and RBBP6 in colorectal cancer, among others [29,30,31].

Moreover, hypermethylation is easily observed at precancerous stages in benign tumors and tumor-predisposing inflammatory lesions [32,33]. In this respect, the retinoblastoma gene’s hypermethylation of the CpG island promoter is released in retinoblastoma [34]. Several research studies have shown promoter hypermethylation and silencing of other TSGs in renal cancer; *VHL* (*von Hippel–Lindau*) [35] in bladder cancer; the cell cycle regulator *CDKN2 A/p16* [36]; and in colon cancer, the mismatch repair gene *hMLH1* [37]. Additionally, abundant hypermethylated TSGs include RASSF10 and SIX3 in kidney cancer and glioblastoma, respectively [38,39]; PTEN, and CDKN2A in melanoma [40,41]; and CDKN2A, TIMPS, and DAPK in prostate cancer [42]. In contrast, significant methylation of EN1 and SCTR was observed in the prostate, colorectal, and salivary gland adenoid cystic carcinoma [43]. The gene APC is hypermethylated in pancreatic cancer [44]. Indeed, hMLH1 still carries a genetic mutation and hypermethylation of an allele in the colon cancer cell line HCT116, which induces the inactivation of the main tumor suppressors [45]. Moreover, pax6, p16, and p15 are generally aberrantly methylated in bladder cancer [46].

### 2.2. Oncohistones and Histone Changes

Histone mutations play an important role in cancer epigenetics, where recurrent mutations targeting histone genes have been described in several types of cancer. Genes coding for histones are mostly mutated in cancer and are commonly named ‘oncohistones’ [47]. Mutations affecting all canonical histone classes and non-canonical histones have been recorded in different tumors [47]. H2A and H2B mutations occur in carcinosarcomas, while H1 mutations were detected in diffuse large B cell lymphomas [47,48]. Similarly, mutations in H3 and its non-canonical counterpart H3.3 were seen in children’s tumors [49,50], whereas mutations in osteosarcoma and giant cell tumor of bone harbor H3.3 G34W mutations [51,52].

Histone modifications control chromatin’s active and inactive states, which ultimately affect gene expression [53]. Important roles are attributed to certain histone modifications in epigenetic acetylation, methylation, and deregulation; linked to epigenetic abnormalities in cancer cells [54,55]. In core histones, H3 and H4, methylation of specific residues and loss of acetylation have been defined as a marker of cancer cells [54,55]. Enzymes that modulate histone modification include histone demethylase (HDMT), histone methyltransferase (HMT), E3-ubiquitin and kinases, histone acetyltransferase (HAT), and histone deacetylase (HDAC) [56].

#### 2.2.1. Histone Methylation and Cancer

Histone methylation affects the affinity of transcription factors, leading to activation or restriction of transcription. Irregularities in the methylation of different lysine residues can modify gene expression [57]. In general, H3K4, H3K36, and H3K79 are the most important sites where their methylation causes gene transcription, while methylation of H3K27, H4K20, and H3K9 is related to transcription silencing [58]. In addition, trimethylations of H3K79, H3K27, and H3K9 induce repression, while mono-methylations of H4K20, H3K27, H3K9, H2BK5, and H3K79 induce gene expression.

H3K27M has been detected in 20% of pediatric glioblastomas and up to 70% of diffuse intrinsic pontine gliomas (DIPGs), resulting in decreased trimethylation of H3K27 (H3K27me3) [50,59,60,61]. Degradation of the EZH2-induced methylation of H3K27 has been reported in breast, prostate, bladder, lung, and kidney cancers and hematological malignancies [62]. Meanwhile, the H3K36M mutation induces undifferentiated sarcoma, causing increased levels of H3K27me3 and global loss of H3K36 (me2 and me) [63,64]. In this regard, H3K27ac enrichment and interactions with other regulatory constituents are promoted by H3K36me2 expansion favors; thus, activating oncogenic pathways [65]. Moreover, increased H3K4me3 is observed in myeloid and lymphoid leukemias [66].

#### 2.2.2. Histone Acetylation/Deacetylation and Cancer

Histone acetylation and deacetylation are correlated with the active and open chromatin conformation and inactive and condensed chromatin form, respectively. In this respect, the HDAC and HAT enzymes control the regulation of histone acetylation, which is very dynamic [67]. The acetyl-CoA acetyl group was added, by HAT, to the histone at the lysine position, which neutralizes the positive lysine charge disrupting the electrostatic interaction between DNA and histones. The chromatin structure was attenuated; thus, affecting the gene assembly and changing the transcription operation [68].

An imbalance of histone acetylation was detected in lung cancers, Rubinstein–Taybi syndrome, AML, and glioblastomas [69]. Many members of HATs mutate differently in tumors [70,71,72] and participate in different stages of its development, including B-cell non-Hodgkin lymphoma and leukemia [73], and have also been described in solid cancers [74], [75]. Furthermore, chromosomal translocations implying HATs and their fusion proteins were involved in the onset and development of acute leukemia [76]. Moreover, any disturbance in the expression of distinct HDAC isoforms induces different cancers. Indeed, dysregulation of HDAC proteins causes aberrant deacetylation and inhibition of TSGs. HDACs can also regulate gene transcription via deacetylating the DNMT, HAT, and HDAC proteins responsible for epigenetic events [77].

#### 2.2.3. Phosphorylation, Ubiquitination, SUMOylation, and Cancer

Abnormalities in kinase activity lead to a variety of cancers [78,79]. The decreased activity of E3 ubiquitin ligase caused by specific mutations can be the origin of various tumors, such as breast cancer and renal cell carcinoma. Moreover, cervical cancer is caused by an increase in the ubiquitination effect, while glioblastoma and colorectal cancer are induced by the total elimination of ubiquitination [80]. SUMOylation is a process required for all cells, and not as a tumor promoter and suppressor [81].

#### 2.2.4. Epigenetic Regulation by miRNAs and Cancer

Epigenetic linked noncoding RNAs (ncRNAs) comprise small interfering RNA (siRNAs), long noncoding RNAs (lncRNAs), microRNAs (miRNAs), and Piwi-interacting RNA (piRNAs). In this regard, ncRNAs are involved in complex double-negative feedback loops, where miRNA inhibition of an epigenetic regulator is performed at the epigenetic level by the same regulator. In contrast, the epigenetic modifier enzymes involved in epigenetic modulation can be targeted by miRNAs, establishing a trilateral regulatory ‘epi–miR–epi’ feedback circuit. The intricate interaction between the epigenetic architecture and miRNAs is important to surveilling gene expression profiles in cancer [82]. They have been classified as tumor suppressors, oncogenic, or context-dependent miRNAs [83]. Furthermore, miRNAs regulate the expression of epigenetic regulatory enzymes, such as HMT, DNMT, and HAT. Moreover, aberrant miRNA profiling indicative of altered regulatory factors, including cell migration and proliferation, has been reported in numerous tumors, with the most manifest being the decreased ncRNA expression levels in cancer cells compared to normal tissues [84].

To date, many aberrant miRNAs have been reported in almost all cancer classes. The first cancer-associated miRNAs were miR-16 and miR-15 in B-cell leukemia [85]. In fact, miR-15-16 and miR-146 are downregulated in cancers, while putative onco-miRNAs such as miR-155, miR-210, miR-221, miR-21, and miR-17-92 are usually upregulated; conferring advantageous traits by targeting TSGs [86]. Additionally, miR-101 and miR-29 target epigenetic modifiers such as EZH2 [87,88] and DNMT3A/B [89], respectively, whereas miR-9 hypermethylation is observed in breast, colon, and pancreas cancers, as well as in acute lymphoblastic leukemia [90,91,92]. The miR-127, which targets BCL6, is negatively methylated and silenced in cancer [93]. On the other hand, the miR-181 family displays inconsistent expression in numerous solid cancers, showing that it could be onco-miRNAs and tumor suppressor miRNAs [94]. Other miRNAs often hypermethylated include miR-92 and miR-29b, which act as oncogenic miRNAs, inducting reactivation of silenced oncogenes [95,96,97].

## 3. The Role of HDAC in Cancer

**Figure 1:**
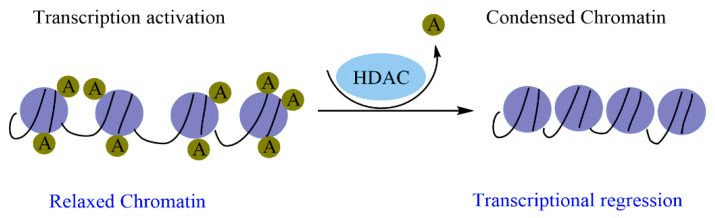
The catalytic action of HDAC enzymes on chromatin condensation.

In many human cancers, dysregulation of epigenetic enzymes results from mutations, abnormal expression, and/or disproportionate recruitment to certain loci. Histone acetylation modulates the chromatin structure, which is a key factor regulating gene expression. Histone acetylation is due to the well-balanced activities of HATs and HDACs. Indeed, HDACs remove the acetyl moiety from lysine residues, resulting in positively charged histones. This increases the ionic interactions with DNA that provoke a compacted chromatin structure, which represses gene expression by making it difficult to access the transcription machinery. Moreover, in the absence of a signal, HDACs can form a corepressor complex with the nuclear receptor and interact directly with transcription factors (Figure 1) [98].

So far, 18 mammalian HDACs have been described and assembled into four families according to their homology with yeast HDACs [99,100]. Homologous to yeast Rpd3, Class I HDACs (1, 2, 3, and 8) are localized in the nucleus of human cells. Class II HDACs (4, 5, 6, 7, 9, and 10) are homologous to yeast Hda1, exhibit a tissue-specific expression, and can shift between the nucleus and cytoplasm. Class III HDACs, or sirtuins (SIRT1-7), are homologous to yeast Sir2 and require the NAD+ cofactor for their activity. Finally, Class IV HDACs, including the recently discovered HDAC11, display the characteristics of both Class I HDACs and HDAC II [99,100].

The implications of HDACs in cancer development were first reported in hematological malignancies by inappropriate involvement of HDAC-containing complexes [100,101]. Until now, very rare mutations altering HDAC expression and activity have been recorded in tumors, while deregulation of their activity has been associated with abnormal gene expression and carcinogenesis. Many studies reported that HDAC1 is overexpressed in prostate, colon adenocarcinoma gastric, and breast carcinomas [102,103,104,105], whereas HDAC2 is overexpressed in colorectal [106], cervical [107], and gastric cancers [108]. Overexpression of HDAC1, HDAC2, and HDAC3 is linked to low survival in patients with gastric and ovarian cancers, while HDAC6 was highly expressed in breast cancer specimens [109]. HDAC8 overexpression was reported in neuroblastoma, whereas low HDAC4 levels are reported in gastric cancers [110,111].

Research findings indicated that knockdown of HDAC genes induced apoptosis and cell cycle arrest, particularly HDAC 1, 2, 3, and 6, in various cancers (colon, breast, lung, and acute promyelocytic leukemia (APL)) [103,111,112,113,114,115]. Knockdown of the HDAC4 gene inhibited cell proliferation and induced apoptosis [116]. Class II HDACs are also involved in angiogenesis regulation, as the knockdown of HDAC6 and HDAC10 reduced angiogenesis-associated VEGFR1 and VEGFR2 [117]. Many reports showed up- or downregulation of SIRT genes in tumors. Along this line, SIRT1 was upregulated in lung cancer [118], prostate cancer [119], and leukemia [59], and downregulated in colon tumors [120]. Embryonic lethality, ascribed to reduced ability to repair DNA damage, was observed in mice lacking SIRT1 [121,122,123]. High levels of SIRT1 in AR-positive prostate cancer cell lines repress their multiplication. Indeed, SIRT1 can elicit senescence and avoid tumorigenesis [124]. SIRT2 is frequently downregulated in human gliomas [124,125,126].

### 3.1. HDAC in Different Cancer Stages

During cancer development, many factors, including inhibition of apoptosis and differentiation, and the promotion of proliferation, angiogenesis, and metastasis are epigenetically regulated. Here, we tried to associate the deregulation of HDACs with different steps of tumor development.

#### 3.1.1. Cell Cycle Progression and Apoptosis

Several HDACs (from 1 to 6) are involved in tumor development, and their loss promotes cell proliferation dysregulation [113,116,127,128,129]. HDAC1 reduces suppressors of the cell cycle mutually with Rb and by altering E2F1 activity [130]. The inhibition of HDAC 1-2 induces cell cycle arrest [131]. HDAC1 is also involved in G_1_/S and G_2_/M transitions. Another study also showed that HDAC1 knock-down contributes to G_2_/M phase arrest [132]. Similarly, both HDAC3 and HDAC10 modulate the G_2_/M transition [133,134]. High levels of Sp1 due to HDAC1/2/6 activities promote the division of cancer cells and G_2_/M progression [135]. Knockdown of HDAC3 induced gathering of cells at the G_2_/M phase [103], whereas in osteosarcoma cells, this effect causes siRNA-mediated HDAC1 depletion [129].

Cell cycle interruption at the G_2_/M stage in renal cancer, following the inhibition of HDAC6 and HDAC3, has been ascribed to proteasomal alteration of Aurora B and A [136]. Furthermore, SIRT1 can suppress the cell cycle through the blockage of p53-dependent pathways [137]. HDAC11 negatively affects E2F7, E2F8, and cell cycle suppressors, leading to survival of tumor cells [138]. Moreover, HDACs act as apoptosis regulators, as the interruption of this process is a critical factor for tumor progression and, therefore, is considered a hallmark of tumor progression. HDACs contribute to the extrinsic, as well as intrinsic, apoptotic pathways. Regarding the extrinsic apoptotic pathways, HDACs can obstruct TRAIL or TGF-b-mediated pathways, while pro- and antiapoptotic factors are altered in the intrinsic pathway [139].

#### 3.1.2. Differentiation

During differentiation, establishing a specific gene expression profile is harmonized by epigenetic modifications, e.g., histone acetylation. In this context, HDAC3 was recruited by RARPML [140], while HDAC4 interacted with RAR-PLZF [141] to repress differentiation-specific transcription. A close mechanism of retinoic acid signaling limitation in hematopoietic cells was recorded in AML1-ETO fusion proteins, which bind to HDAC1, 2, and 3 [142,143]. HDAC8 is a key regulator of cancer cell differentiation [144], and HDAC8 overexpression is associated with neuroblastoma progression.

#### 3.1.3. DNA Damage Response

HDACs contribute to DNA damage repair (DDR) responses via their key role in remodeling chromatin and regulating the acetylation patterns of proteins associated with DNA [145]. The inhibition of HDAC blocks double-strand break (DSB) repair and radio-sensitizes cancerous cells. In this line, HDAC2 and HDAC1 bind to DNA damage regions, to deacetylate histones at H3K56 and H4K16 and promote non-homologous end-joining pathways, which accelerate DSB repair [146]. Moreover, HDAC3 is involved in nucleotide excision repair (NER) [147], whereas HDAC9 and HDAC10 contribute to homologous recombination [148,149]. HDAC6, in association with DNA mismatch repair protein (MSH2), acts as an MSH2 inhibitor through deacetylation and ubiquitination [150]. In addition, Sirtuins interact with numerous proteins regulating several DDR pathways [151]. In tumor cells, SIRT1 restraints p53 acetylation, contributing to cell survival [148,152]. SIRT6 phosphorylation is directly engaged in DNA damage sites to promote DSB repair [153,154]. In leukemia-initiating cells, the inhibition of SIRT6 or HDAC8 engenders a DNA repair deficit in homologous recombination and the NHEJ pathway [155].

#### 3.1.4. Metastasis

The capacity to disperse and metastasize represents the deadliest signature of tumor cells. Numerous works have evidenced that HDACs regulate metastasis in various cancers. An important player in metastasis is the transition from adherent epithelial cells to motile mesenchymal cells capable of leaving the primary tumor site. In embryonic development, EMT is a crucial path for cell migration during gastrulation [156]. In colorectal cancer, HDAC3 was engaged to Runx 2 promoter and hampered metastasis [157]. Research findings showed that HDAC7 enhances EphA2 expression by downregulating miR-4465 expression, positively affecting tumor proliferation, spread, and invasion in nasopharyngeal cancer [158]. Similarly, HDAC11 leads to the overexpression of RRM2, a gene involved in promigratory and metastatic phenotypes [138]. In prostate cancer, ZEB1 and SIRT1 together bind to CDH1, promoting metastasis [159], while SIRT1 elicited EMT through Fra-1 over-expression in colorectal cancer [160].

Nonetheless, SIRT1 seems to limit metastasis, not only via deacetylation of Smad4, but also via repression of TGF-b-triggered signaling, whereas SIRT2 deacetylated Slug and stabilized its protein to increase EMT [161]. Many metastasis repressors, such as RECK, Kangai 1, RhoB, and TIMP-1, increased their level in response to HDAC inhibitors, indicating a potential role of HDACs in the promotion of metastasis [162]. In addition, HDAC1 impaired the expression of RECK and RhoB in cancer cells [163]. EMT is promoted by transcription factors of the SNAIL/SLUG family, mediating the silencing of E-Cadherin expression by engaging the SIN3/HDAC1/HDAC2 corepressor complex and upregulating the matrix metalloproteinase family proteins. Furthermore, SNAIL1, by interacting with HDAC1-2, inhibits the E-Cadherin promoter, since the addition of trichostatin A (TSA) obstructs the effect of SNAIL1 in SNAIL1-expressing epithelial cells [164]. The pivotal role of SNAIL1 in association with HDAC1/HDAC2 and resulting in E-Cadherin repression in cancers has been documented [165].

#### 3.1.5. Angiogenesis

Angiogenesis involves the creation and addition of new blood vessels and is pivotal for the development of tumors [166,167]. The first steps of angiogenesis are elicited by hypoxia or a hypoxic microenvironment, while its advancement is mainly controlled by hypoxia-inducible factor 1a (HIF-1a). Overall, HDACs monitor the balance between pro- and anti-angiogenic proteins. In this context, HDAC inhibition exerts anti-angiogenic activity via inhibition of pro-angiogenic gene expression. Under hypoxia conditions, Class I HDACs, mRNA, and protein were overexpressed in vitro in primary and malignant cells [168]. HDAC1 deacetylates HIF-1a, contributing to preventing HIF-1a loss. On the other hand, dysregulated levels of HDAC1 lead to high levels of HIF-1a and VEGF in tumors, which in turn enhances angiogenesis [169]. HDAC4, 6, 10, and SIRTs display similar pathways [139], whereas HDAC4, 5, and 6, acting as mediators of HIF-1 activity, require cofactors (HSP90 and p300) [170]. On the contrary, it was shown that SIRT1 deacetylated HIF-1a, which reduces the interaction of HIF-1a with p300, which reduces HIF-1a activity. In endothelial cells, HDAC5 reduced the expression of pro-angiogenic genes (FGF2 and Slit2) [171]. Additionally, HDAC5 represses cysteine-rich angiogenic inducer 61 (CYR-61), a well-known antifibrotic and pro-angiogenic mediator, inhibiting angiogenesis [172]. HDAC6 enhances angiogenesis via deacetylation of cortactin, an actin-remodeling protein [173].

#### 3.1.6. Autophagy

Autophagy is a process that suppresses damaged subcellular fractions, helping to intercept the transformation of normal cells to cancerous ones [174,175]. Published data showed that Class I HDACs mediate autophagic flux in mice [50], whereas elimination of HDAC1 and HDAC2 impedes autophagic flux [176]. On the other hand, HDAC4 and HDAC5 influence autophagic flux by acting as positive regulators of tumor cell development. HDAC6 promotes autophagy, based on its connection with microtubule proteins [177,178]. In this regard, autophagy might be actively enhanced and play a compensatory role for HDAC6 under ubiquitin-proteasome system damage [120,148]. At the same time, HDAC6 shows a significant role in ubiquitin-selective quality control autophagy, instead of starvation-induced autophagy [163]. Similarly, Parkin-mediated mitochondrial ubiquitination could engage the autophagic actors, i.e., HDAC6 and p62 [179]. HDAC10 knock-down induces autophagosome/lysosome fusion blockade and restriction of autophagic flux, which sensitizes cells to chemotherapy [180]. HDAC10 also deacetylates HSP70 protein members associated with autophagy-mediated cell longevity [180]. In contrast, SIRT1 displays a dual role in autophagy [181], where it is necessary to trigger starvation-induced autophagy [127,182]. Moreover, SIRT1 deacetylates forkhead box O3 (FOXO3), leading to proteasomal degradation and, thus, contributing to the overexpression of numerous autophagic genes.

In embryonic stem cells (ESCs), SIRT1 affects the PI3K/Beclin 1 and mTOR pathways, affecting oxidative stress-induced autophagy [183]. SIRT2 detaches from FOXO1 under stress conditions, which promotes hyperacetylated FOXO1, promoting the autophagic process [184]. In contrast, deacetylation of lactate dehydrogenase B (LDHB) by SIRT5 intensifies its effect. Protons (H^+^) generated by LDHB promote autophagy in tumor cells [181,185]. Furthermore, SIRT5 is involved in ammonia-induced autophagy, via glutamine metabolism remodeling [184]. SIRT6 promotes autophagy by hampering the transcriptional repressor Nkx3.2, resulting in the expression of GATA5 [186].

## 4. Natural Bioactive Compounds Targeting HDAC in Human Cancers

### 4.1. Flavonoids

#### 4.1.1. Epigallocatechin Gallate

**Figure 2:**
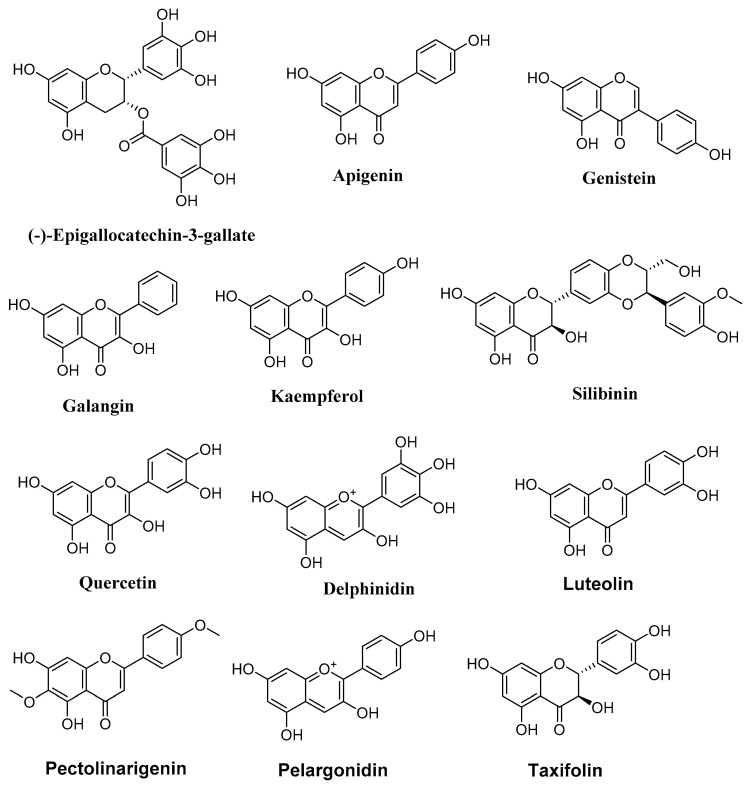
Chemical structures of flavonoids with anticancer activity by targeting HDAC.

Epigallocatechin gallate (EGCG) (Figure 2, Table 1), a flavonoid found in green tea, exhibits potent anticancer properties, by targeting epigenetic mechanisms, especially HDAC pathways. Published research showed that EGCG reduced the expression levels of HDAC2 and HDAC3, by 50 μM to 150 μM for 48 and 72 h [187], as well as downregulating the HDAC1 protein level by approximately 50% at 100 μM [188] in human colon cancer cell lines. Treatments with EGCG (40 μmol/L) and a pro-drug EGCG (pEGCG) (20 μmol/L) induced a significant suppression in the proliferation of both MCF-7 and MDA6MB 231 breast cancer cells via epigenetic mechanisms, as it inhibited the activities of histone acetyltransferase (HAT) [189]. This compound can remodel the chromatin structures of the *hTERT* promoter by downregulating acetyl-histone H3 and H4, as well as acetyl-H3K9 expression levels, accompanied by chromatin alterations, leading to the binding of different hTERT repressors, including E2F-1 and MAD1 to the hTERT regulatory region [189]. In skin cancer A431 cells, EGCG (25 μM) has been reported to induce an increase in acetylated lysine 14 and 9 (on histone H3) and acetylated lysine 12, 5, and 16 on histone H4 (H3-Lys and H4-Lys) for six days [190]. Recently, it was found that EGCG (200 μM) can increase H3K9/14ac and H3ac histone acetylation up to 8-fold compared to the reference, accompanied by concomitant hypermethylation of the active H3K4me3 (almost 2.5-fold) and the repressive H3K9me3 chromatin proteins (up to 160%), correlating with downregulation of heterochromatin binding factors such as HP1α and HP1γ [191].

#### 4.1.2. Apigenin

Apigenin (4′,5,7-trihydroxyflavone) (Figure 2, Table 1) is a yellow crystalline solid flavone with several natural sources, including medicinal plants. In human prostate cancer PC-3 cells, apigenin (20–40 μM) acts as a potent HDAC inhibitor, which induces noticeable suppression of the HDAC protein activity (41% and 62%), especially HDAC1 and HDAC3 [192]. It additionally promoted global acetylation of both histone H3 (7.4- and 8.2-fold) and H4 (1.2- and 2.6-fold), as well as specifically centered the hyperacetylation of histone H3 on the p21/waf1 promoter [192]. In this context, Tseng et al. [193] demonstrated that apigenin treatment for 48 h could inhibit the HDAC activity in breast cancer cells. Moreover, molecular analysis revealed that apigenin increases the expression levels of acetylated H3, particularly in the p21^WAF1/CIP1^ promoter area mediated by the upregulation of p21^WAF1/CIP1^ transcription [193].

#### 4.1.3. Galangin

Galangin (Figure 2, Table 1) is a natural flavonoid found in honey and in lesser galangal (*Alpinia officinarum* Hance.). This natural compound exhibits a promising effect against HDAC activity. Treatment with galangin for 24 h increased endogenous HDAC1-mediated deacetylation, independent of DNA methylation status, and subsequently decreased histone H3 acetylation in the BACE1 promoter regions in human neuroblastoma SH-SY5Y cells [194].

#### 4.1.4. Genistein

Genistein (Figure 2, Table 1) is a naturally occurring isoflavone, first isolated from *Genista tinctoria*. This compound is known for its promising chemotherapeutic effect against several types of cancer. Genistein (5–20 μmol/L) showed, in vitro, a weak inhibitory capacity of HDAC catalytic activity (13–17%) in human esophageal squamous carcinoma cells [195]. HAT and HDAC analysis demonstrated that genistein decreases HDAC, but increases HAT activity. ChIP analysis with various antibodies showed enrichment of acetylated histones H3, H4, and H3 di- and tri-methylated lysine 4 in prostate carcinoma cell lines (LNCaP, PC3) [196]. On the other hand, using mouse models, Li and coworkers [197] reported that a diet containing 250 mg/kg genistein reduced HDAC1 expression in vivo at protein and mRNA levels. Moreover, genistein also suppressed the phosphorylation of serine ten and methylation of lysine nine at the promoter regions of various genes, such as *Wnt5a*, *Sfrp5*, and *Sfrp2* [198].

#### 4.1.5. Silibinin and Kaempferol

Silibinin (Figure 2) is a flavonolignan derived from milk thistle, *Silybum marianum*, and has potent anticancer properties, targeting various checkpoints, including epigenetic mechanisms such as HDAC activity. Research findings demonstrated that silibinin (10–75 μM) downregulates the levels of HDAC2 and HDAC3 proteins, as well as HDAC1, HDAC6, SET domain proteins (SETD1A, D4, D6), and lysine-specific demethylases (KDM 5B, 5C, 4A) in non-small lung cancer H1299 cells [199]. In human prostate cancer cell lines (DU145 and PC3), silibinin (25–75 μg/mL) decreased the expression levels of HDAC1-2 for 48 h in a concentration-dependent manner [200]. It also increased trimethylation levels of Lys27 on H3 (H3K27me3) [200]. On the other hand, kaempferol (5 μM) has been reported to exhibit epigenetic modification in human hepatoma HepG2, Hep3B, and HCT-116 cells by binding to human HDAC enzymes and sequentially induced hyperacetylation of the histone complex H3 [201].

#### 4.1.6. Quercetin

Quercetin (Figure 2) can be found in medicinal plants and numerous food products. This compound possesses different biological activities, including anticancer, via various mechanisms of action. Quercetin alone, or combined with other compounds, exhibited effective epigenetic modifications. At a concentration of 100 μM, it increased the histone H3 acetylation mediated by the upregulation of FasL expression, and activated the HAT and inhibited the HDAC activities [202]. In addition, quercetin (25 and 50 μM) modulated the expression of numerous chromatin modulators after 48 h of treatment and decreased HDAC and HMT activities, including the activity of HMT-H3K9 [203]. Listed in Table 1 are the natural flavonoids targeting HDAC in human cancers.

### 4.2. Alkaloids

**Figure 3:**
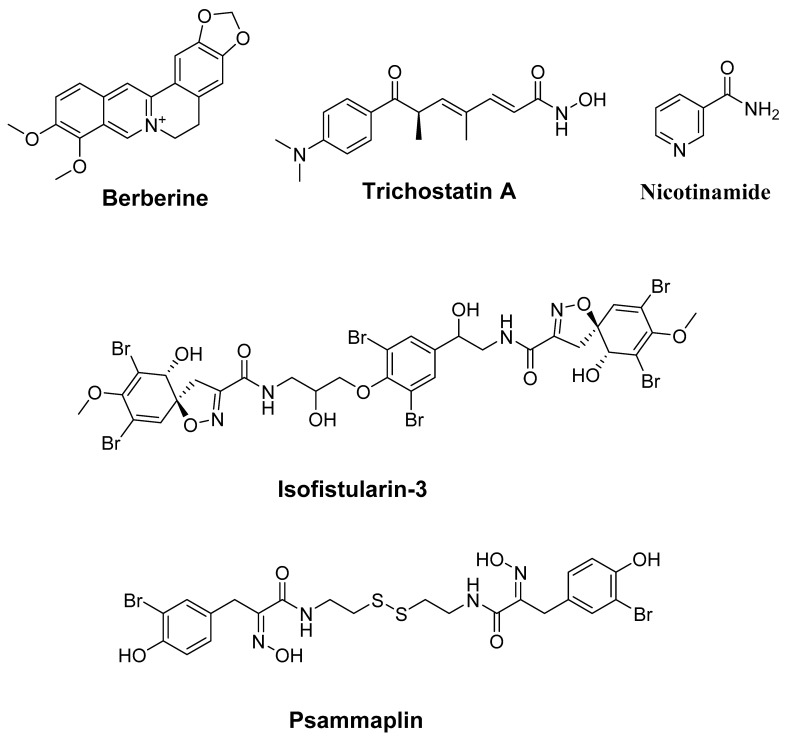
Chemical structures of natural HDAC alkaloids in human cancers.

Alkaloids such as berberine, nicotinamide, isofistularin-3, psammaplin, and TSA (Table 2, Figure 3) exhibit promising anticancer effects via the control of epigenetic changes and various other mechanisms. The HDAC signaling pathways represent some of the main targets of these bioactive compounds. In this context, berberine (120 μM) exhibits remarkable anticancer effects via targeting epigenetic chromatin modification, which affects the enzyme implicated in histone acetylation and methylation in acute myeloid leukemia (AML) cell lines [224]. These events revealed the role of berberine as a master epigenetic regulator responsible for controlling downstream bioactivities, including cell cycle arrest, apoptosis, and the underlying mechanism of metabolic disorders and immune response.

The findings by Florean and colleagues [225] revealed that isofistularin-3, a brominated alkaloid derivative isolated from *Aplysina aerophoba,* can inhibit DNMT1 in vitro (IC_50_ = 13.5 ± 5.4 µM), through binding to the DNA-interacting pocket of the enzyme. In addition, Iso-3, at a dose of 25 µM, induced modification of the *AHR* promoter methylation and upregulated the AHR expression in RAJI cells. These effects are probably associated with generating an altered chromatin state via histone deacetylase activity (HDAC). These results were validated in vivo in a zebrafish xenograft model. Notably, researchers provided evidence that Iso-3 does not elicit acute toxicity in developing zebrafish, shown by the absence of morphological defects and dead embryos, even at the highest dose, after 24 h of treatment. Moreover, using a mice skin model, Tiwari and Gupta [226] found that nicotinamide (NA) treatment (5 mg) increases the expression levels of HDACs, mediated by the downregulation of tumor suppressor miR-203, at a period between 4 and 16 weeks. The combination of this molecule (NA) with 7, 12-dimethylbenz [a] anthracene, a potent carcinogen compound, exhibited further effects: targeting the HDAC pathway, providing a novel chemoprevention strategy by combining different compounds through the control of epigenetic and microRNA biogenesis modulators in tumor development, in a time-dependent manner [226]. Within this context, the study of Vincent et al. [227] demonstrated that TSA (0.3 µM), a hydroxamic acid first identified in the bacterium *Streptomyces platensis*, exhibits a broad-spectrum of reversible HDAC inhibitory activity. Chromatin immunoprecipitation and RNA interference assays showed that TSA inhibits the histone deacetylation concomitant with potent *MUC4* repression in high-expressing cells [227]. Moreover, treatment of breast MDA-MB-231 cell lines with TSA (100 ng/mL for 12h) combined (25 μM for two days) with genistein showed promising histone changes in the estrogen receptor-α (ERα) promoter and consecutively decreased the HDAC activity [197]. The potential toxicity of this new combination has been reported in normal human mammary epithelial cells (HMECs). It has been shown that the combined treatment of TSA with genistein is potentially safe and can be applied to in vivo experiments.

In a similar fashion, Ahn and coworkers [228] reported the antiproliferative activity (IC_50_ = 5μg/mL) of the alkaloid psammaplin A as an HDAC suppressor. They showed that this molecule exhibited remarkable epigenetic regulation and exhibited antiproliferative activity against endometrial cancer cells at the lowest dose of 5 μg/mL, triggering genes related to apoptosis and cell cycle arrest, accompanied by accumulation of acetylated histones and decreased expression levels of HADC [228]. Moreover, using human cancer cell lines, Baud et al. [229] reported the molecular and the enzymatic mechanisms of psammaplin A against its HDAC targets. These researchers showed that psammaplin A (11c) remarkably affects HDAC1 in vitro (IC_50_ = 0.9 nM). Regarding the enzymatic specificity of psammaplin A, it has been found that this compound exhibits strong isoform selectivity, which is 360-fold selective for HDAC1 compared to HDAC6, and more than 1000-fold less effective against HDAC8 and HDAC7.

### 4.3. Terpenoids

#### 4.3.1. Corosolic Acid

**Figure 4:**
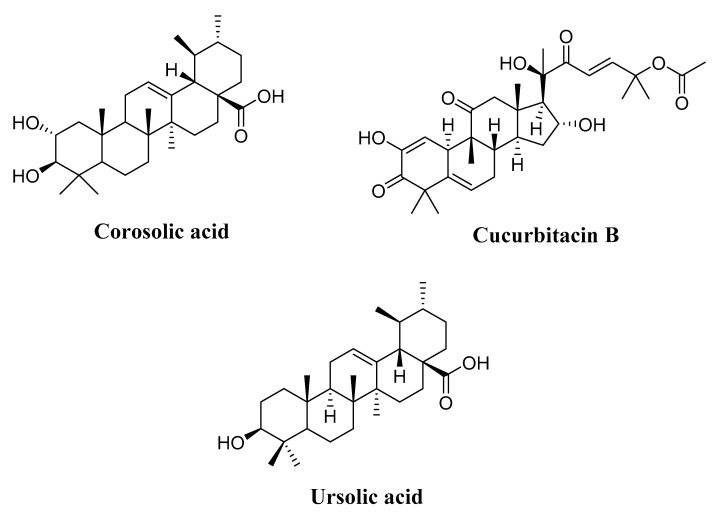
Chemical structures of HDAC natural terpenoids in human cancers.

Corosolic acid (Figure 4, Table 3) is a triterpene acid isolated from *Lagerstroemia speciose*. This bioactive compound is commonly abundant in food sources such as guava, loquat, and olive, and exhibits various pharmacological effects, including anti-proliferative activity. In this respect, Zhang and coworkers [232] studied the role of corosolic acid in the activity and expression of HDACs, in a transgenic adenocarcinoma mouse prostate (TRAMP) model, as well as its involvement in DNA and histone H3 methylation. These researchers found that corosolic acid promotes downregulation of the expression and activity of epigenetic modulatory proteins, demonstrating its ability to prevent prostate carcinogenesis. In addition, corosolic acid (4 μM) significantly increased the expression levels of the acetylated histone H3 lysine 27 (H3K27ac), accompanied by a decrease in histone H3 lysine 27 trimethylation (H3K27Me3) at *Nrf2* promoter in TRAMP-C1 cells [232].

#### 4.3.2. Cucurbitacin B

Cucurbitacin B (Figure 4) is a plant-derived triterpene, initially found in the cucurbitaceae plant species. Published research has indicated that this molecule acts as a potent HDAC inhibitor at sub-IC_50_ and at IC_50_s in the nanomolar range (IC_50_ = 60 nM) for non-small cell lung cancer cells (NSCLC H1299 cells). Cucurbitacin B induced suppression of the activity and expression levels of epigenetically modifying marks. It also produced histone changes at the *p16^INK4A^*, *p21^CIP1/WAF1^*, and *hTERT* promoters in human NSCLC H1299 cells [233]. An in vivo experiment using a mouse model for lung cancer showed that cucurbitacin B at a concentration of 0.2 mg/kg b.w significantly inhibits the expression levels of different HDAC proteins (1–4), without visible signs of toxicity in animals.

#### 4.3.3. Ursolic Acid

Ursolic acid (3-beta-3-hydroxy-urs-12-ene-28-oic-acid) (Figure 4) is a triterpenic acid distributed in different plants and foods, such as ginseng (*Panax Ginseng*), rosemary (*Rosmarinus officinalis*), apple peel, pear, cranberry, and plum (*Prunus domestica*) [234,235]. It has been widely explored for its chemopreventive and chemotherapeutic effects on various cancers. Ursolic acid has been investigated for its action against different epigenetic regulators, including HDAC proteins. Research findings showed that this bioactive compound considerably downregulates the expression of several epigenetic modulatory factors, starting at a dose of 2.5 μM (non-toxic concentrations), including HDAC1, HDAC2, HDAC3, and HDAC8 (Class I), in addition to HDAC6 and HDAC7 (Class II) [232]. Ursolic acid also suppressed induction of HDAC1 and HDAC3 markers in leukocytes, mediated by LPS in mouse epidermal cells [232].

### 4.4. Fatty Acids

#### 4.4.1. Butyrate

Butyrate is a short-chain fatty acid, mainly generated during the fermentation of carbohydrates by the intestinal microflora [242]. This molecule exerts an efficient epigenetic effect, acting as an HDAC inhibitor (Table 4). In this respect, Saldanha and coworkers [206] reported that butyrate induces important epigenetic modifications at relatively high, but physiologically achievable, concentrations (5 mM), as it affects the global DNA methylation and chromatin structure; thus, inhibiting HDAC1 expression in RKO, HCT-116, and HT-29 colorectal cancer cell lines. Moreover, butyrate decreased the reverse expression of HDAC1 in human esophageal 9706 cancer cells [223]. The combination of butyrate with quercetin showed the reverse effect, triggering remodeled DNA methylation and histone acetylation; thus, inhibiting HDAC activity [223]. This effect was mediated by NF-κB epigenetic signaling cascades. Generally, butyrate, through its epigenetic regulatory effects on HDACs, exhibits functions as a modulator of chromatin structure and may mediate cellular responses via up-regulatory genes responsible for cell cycle arrest, promoting therapeutic outcomes.

#### 4.4.2. Butyric Acid and Eicosapentaenoic Acid

Butyric and eicosapentaenoic acids are fatty acids with several vegetable sources and exhibit important biological properties, including anticancer activity with different mechanisms, such as their effect on epigenetic targets (Table 4). Tiwari and Gupta [226] reported, in vivo, the antitumor activity of butyrate alone (17.62 mg) or combined with other compounds, including nicotinamide (NA) (5 mg) and calcium glucarate (CAG) (5 mg). The results showed that butyric acid exhibits a promising effect on 7,12-dimethylbenz[a]anthracene (DMBA)-induced tumor through increasing HDAC expression and activity, accompanied by upregulation of miR-203 promoter methylation at 4 or 16 weeks [226]. This effect was intensively promoted by co-administration of butyric acid with NA and CAG, targeting epigenetic or biogenetic modulations. On the other hand, treatment with eicosapentaenoic acid (100 μM) promoted the expression of the tumor suppressor gene by downregulating the expression levels of the HDAC1 enzyme, mediated by the activation of peroxisome proliferator-activated receptor (PPARγ) in hepatocarcinoma cells (HCC).

### 4.5. Isothiocyanate

#### 4.5.1. Phenethyl Isothiocyanate (PEITC)

**Figure 5:**
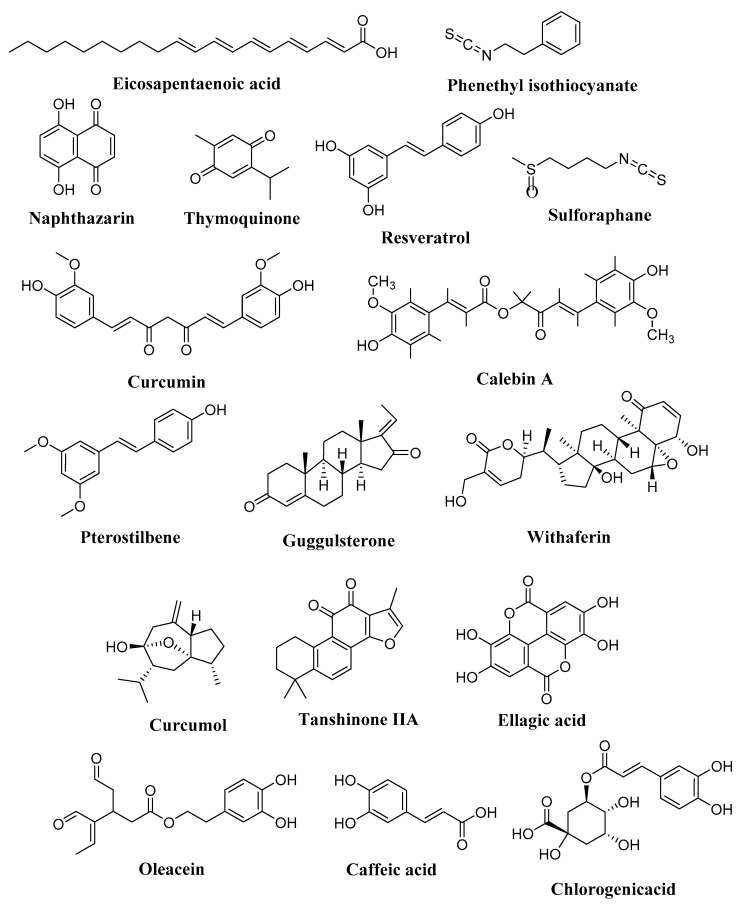
Chemical structures of different compounds associated with HDAC in human cancers.

Phenethyl isothiocyanate (PEITC) (Figure 5, Table 5), is a secondary plant metabolite, naturally present as gluconasturtiin in numerous cruciferous vegetables, and has shown remarkable anticancer properties via multiple mechanisms, including as an epigenetic regulator. In one study, PEITC was used at multiple doses (0–20 μM). Long-term PEITC treatment (6 weeks; 2.5 μM) resulted in a 40% reduction in viability compared to cells exposed to the DMSO vehicle alone, but similar effects were not evident in a culture of 72 h, demonstrating that 2.5 μM PEITC is not highly cytotoxic in short-term cultures [244].

Several HDACs, including HDAC1, HDAC3, SAP18 (sin3-associated protein 18), and SAP30 (sin3-associated protein 30), showed preferential attachment to heterochromatin after exposure to PEITC, indicating that PEITC may prevent HDAC binding from opening areas of active euchromatin. On the other hand, short-term PEITC treatment had very minor impacts on the distribution of histone epigenetic regulators in SW620 cells, suggesting that long-term exposure is required to generate persistent and heritable alterations in tumor cell epigenetic profiles [244]. In human colon carcinoma cell line HT29, PEITC demonstrated a cytotoxic effect with an IC_50_ of 11.88 μM [245]. Further in vivo investigation using PEITC indicated that a daily dose of 60 mg/kg p.o for 21 days could inhibit HDAC overexpression in rats [245].

#### 4.5.2. Sulforaphane (SFN)

Sulforaphane (1-isothiocyanato-4-(methylsulfinyl)butane) (Figure 5) is an isothiocyanate mainly distributed in diverse cruciferous vegetables, such as broccoli, cabbage, brussel sprouts, and radish [246]. Treatment with SFN (15 μM and 20 μM) for six days decreased the growth of MCF10A cells, suggesting that these concentrations may be hazardous to normal breast cells. Breast cancer cells are preferentially inhibited at doses of 10 μM or less. Sulforaphane therapy at a dose of 10 μM was shown to dramatically suppress HDAC activity in breast cancer cells [247]. Similar events were also noticed in A549 and H1299 lung cancer cells, where SFN significantly inhibited HDAC activity and upregulated the expression levels of acetylated histones H3 and H4 [248]. On the other hand, SFN increased the anti-oncogene proteins DUSP4 and CDK expression, which correlated with the downregulation of HDAC5 and HDAC11 genes in the hepatocarcinoma HepG2 cell line [249]. Listed in Table 5 are natural compounds that target HDAC in human cancers.

### 4.6. Quinones

#### 4.6.1. Naphthazarin

Naphthazarin (5,8-dihydroxy-1,4-naphthoquinone) (Figure 5) is a naturally occurring bioactive compound that shows various biological effects, such as antioxidant, antiparasitic, antifungal, and anticancer activities [247,248]. The viability of MCF-7 cells treated with naphtarazin was reduced in a dose-dependent manner (0–5 μM). Real-time PCR findings revealed that naphtarazin reduced HDAC1 mRNA expression in a dose-dependent manner [256]. This molecule inhibited the expression of HDAC1, resulting in increased expression levels of p21 cell cycle inhibitor, accompanied by a reduction in the expression of the chromatin effector proteins UHRF1 in MCF-7 [256].

#### 4.6.2. Thymoquinone

Thymoquinone (TQ) (Figure 5) is the main bioactive compound of the black seed, *Nigella sativa* L. It possesses various health benefits and biological activities, including anticancer, anti-inflammatory, antiviral, antioxidant, antiparasitic, antimicrobial, immunomodulatory, and anticoagulant properties [250,251]. Moreover, TQ at doses of 5, 10, and 20 µM decreased cell proliferation and the expression of *UHRF1, DNMT1, G9a*, and *HDAC1* genes in Jurkat cells and MDA-MB-468 cells [257].

### 4.7. Stilbenes

#### 4.7.1. Resveratrol

Resveratrol (3,5,4′-trihydroxystilbene) (Figure 5) is a bioactive compound, first extracted from the roots of white hellebore (*Veratrum grandiflorum* Loes.) by Saiko et al. [276]. This bioactive compound is found in more than 50 different plant species, including grapes, apples, blueberries, plums, and peanut. It has been extensively explored for its health benefits against diverse pathologies, including cancer [277]. The cytotoxic evaluation of resveratrol against HeLa, SiHa, and Caski cells revealed that the IC_50_ values were 17, 22, and 118 µM, respectively [259]. For Caski cells, treatment with resveratrol for four days increased *p21* expression by 1.7-, 2.1-, and 28-fold, respectively, compared to control cells (HDAC inhibition is characterized by a rise in *p21*, which is controlled by *UHRF1*) [259].

The translocation of B16F10 tumor cells to the lungs was dramatically reduced after 20 days of intraperitoneal (50 mg/kg) resveratrol therapy in 7-week-old female mice. Resveratrol showed no cytotoxicity against B16F10 cells at a dose of 60 μM, and it was speculated that resveratrol is involved in its anti-migration function, since HDAC1 and ACAT1 are resveratrol targets [260]. Similarly, resveratrol treatments, even at low doses (10 μM), reduced the cell viability of MCF-7 and MDA-MB-231 cell lines in a dose-dependent manner. Using increasing amounts of resveratrol (0–100 μM), researchers found that it suppresses HDAC activity in both cell lines in a dose-dependent manner [278].

#### 4.7.2. Curcumin

Curcumin (Figure 5) has been reported to be a potent epigenetic regulator that has multiple effects on HDAC expression and activity. Curcumin downregulated the expression levels of HDAC1, HDAC3, and HDAC8 proteins and histone acetyltransferase p300, increasing the Ac-histone H4 protein expression in Raji cells. Curcumin was used at different concentrations (6.25, 12.5, 25, 50, and 100 μmol/L). Compared to curcumin-treated cells (25 μmol/L for 24 h), the expression of HDAC1, HDAC3, and HDAC8 was considerably higher in Raji cells. When cells were treated with 25 μmol/L curcumin for 24 h, more than two-thirds of them could not multiply. The IC_50_ for 24 h was 25 μmol/L [263]. Curcumin was found to inhibit histone acetyltransferase (HAT) activity in LNCaP cells at a concentration of 5 μM, and it has also been suggested to be a possible DNMT and HDAC inhibitor [267].

#### 4.7.3. Calebin-A

Calebin-A (Figure 5) is a curcuminoid compound obtained from *Curcuma longa* roots. This molecule possesses various biological properties [279]. It inhibited the proliferation of the malignant peripheral nerve sheath tumor (MPNST (STS26T, ST8814, T265, and S462TY)). At concentrations of 12.5–25 μM, Calebin-A reduced the viability of MPNST cell lines. With a 25 μM dose of Calebin-A applied for 24 h; all MPNST cell lines had survival rates below 50%. On the other hand, HDAC activity in MPNST cells did not decrease significantly after treatment [268].

#### 4.7.4. Pterostilbene

Pterostilbene (3,5-dimethoxy-4-hydroxystilbene) (Figure 5) is a naturally occurring bioactive compound found in grapes and numerous berries, especially blueberries. Chemically, pterostilbene is a phytoalexin dimethyl ether compound derived from resveratrol [280]. Combinatorial treatment of pterostilbene with resveratrol (5 μM) modulated gene expression in HCC1806 and MDA-MB-157 breast cancer cells through epigenetic processes, including modification of HDACs [269]. It induced a downregulation of SIRT1, a type III HDAC, which is involved in modifying histones and some non-histone proteins via deacetylation, and subsequently modulates cell proliferation, apoptosis, stress response, metabolism, cellular senescence, and tumorigenesis [269,281].

### 4.8. Steroids

#### 4.8.1. Guggulsterone

Guggulsterone [4, 17(20)-pregnadiene-3, 16-dione] (Figure 5) is a phytosteroid compound isolated from the gum resin of *Commiphora wightii*. This compound has been shown to prevent and treat numerous types of cancers. In a study by Mirza et al., the IC_50_ of guggulsterone for MCF 7 cells was 20 μM and for MDA MB 231 cells 15 μM, and at low concentrations, guggulsterone induced a remarkable effect on the modulation of key epigenetic regulators, such as HDACs responsible for the activation of tumor suppressor genes. Moreover, it has been reported that this bioactive compound can reverse the epigenetic modifications resulting from DNA hypermethylation, via suppressing HDAC1 expression in both human breast cancer MCF7 and MDA-MB-231 cell lines.

#### 4.8.2. Withaferin A (WFA)

Withaferin A (Figure 5) is a steroidal lactone extracted from the *Withania somnifera*, known for its antitumor properties, and targeting different hallmarks of cancer, including cell proliferation, migration, invasion, and angiogenesis, as well as the epigenetic process. WFA demonstrated chemopreventive effects against breast cancer, reversing the epigenetic changes via downregulation of HDAC1 protein levels in MCF7 and MDA-MB-231 cell lines. The IC_50_ values for MCF 7 and MDA MB 231 cells were 8 and 10 μM, respectively. Downregulation was also observed in HDAC1 expression in MDA MB 231 cells. In another study, WFA alone or combined with SFN (at 1 and 5 μM, respectively) exhibited remarkable downregulation of HDAC1 expression at both mRNA and protein levels in the MCF7 and MDA-MB-231 cells, with a significant effect using combination therapy [250]. WFA with SFN also reduced HMT activities; however, they increased HAT activities, especially against MDA-MB-231 cells [250].

### 4.9. Phenolic Acids, Secoiridoids, Tannins, and Tanshinones

#### 4.9.1. Phenolic Acids: Caffeic Acid Chlorogenic Acid

Caffeic acid and its derivative chlorogenic acid (Figure 5) are phenolic acids mainly found in numerous plants and have shown promising health benefits against several diseases. These two compounds are effective epigenetic agents, inhibiting HDAC activity. Using an in vitro HDAC assay, Bora-Tatar and co-workers [265] showed that chlorogenic acid exhibits potent inhibition of HDAC activity (IC_50_ = 375 μM). In contrast, moderate inhibition was observed by caffeic acid (IC_50_ = 2.54 mM).

#### 4.9.2. Secoiridoids: Oleacein

Oleacein (Figure 5) is the most abundant phenolic compound of *Olea europaea* L. (olive), belonging to the secoiridoid class. This compound has shown to be an efficient epigenetic modulator on multiple myeloma cell lines (NCI-H929, RPMI-8226, U266, MM1s, and JJN3). In fact, oleacein was shown to reduce cell viability in a dose-dependent manner, 48 h after treatment, with IC_50_s ranging from 5.0 to 20.0 μM. On the other hand, this molecule did not affect the vitality of PBMCs from healthy donors. Unlike the positive controls, TSA and SAHA, incubation with oleacein had no effect on the HDAC activity retrieved from nuclear extracts. These data imply that oleacein does not affect MM cell acetyloma through enzymatic HDAC suppression [270].

#### 4.9.3. Secoiridoids: Ellagic Acid

Ellagic acid (EA) (Figure 5) is a ubiquitous phenolic compound extracted from various fruits and vegetables, and which is well-known for its bioactivity against different cancer cells. This compound has demonstrated effective epigenetic HDAC modification. A significant increase in the expression of the HDACs gene was observed in human adipogenic stem cells treated with EA (twenty-fold higher than the control). Furthermore, 10 µM EA inhibited HDAC9 downregulation after four days of cell treatment. This phenolic compound also reduced adipocyte development and differentiation by inducing histone arginine methylation, and thereby increased acetylated histone through epigenetic changes, mediated by coactivator-associated arginine methyltransferase 1 (CARM1) inhibition. The findings indicated that CARM1 inhibition induces suppression of H3R17 methylation, resulting in decreased H3K9 acetylation and HDAC9 dissociation [271].

#### 4.9.4. Tanshinones: Tanshinone IIA

Tanshinone IIA (Figure 5) is a natural bioactive compound identified in the rhizome of *Salvia miltiorrhiza* Bunge. Wang et al. [272] investigated the role of tanshinone IIA in epigenetic pathways, illustrating its effect on HDAC modification. These researchers showed that this bioactive compound inhibited the enzymatic activity of HDACs by 50% in the tanshinone treatment concentration range of 5.0 to 10.0 μM. Tanshinone IIA also downregulated, in a concentration-dependent manner, HDAC1, HDAC3, and HDAC8 protein levels, by decreasing mRNA expression [272].

### 4.10. Other Molecules Targeting HDAC in Human Cancers

#### 4.10.1. Arsenic Trioxide

Arsenic trioxide (As_2_O_3_), a naturally occurring inorganic compound and environmental pollutant, is considered a potent human carcinogen [273,282]. This compound has demonstrated an effective anticancer activity, acting on epigenetic regulators (Table 5); it increased HDAC4 expression in human HeLa and HEK293T cell lines [273]. Treatments conducted for 24–72 h in the concentration range of 0.2–0.8 μM showed that As_2_O_3_ induced histone H4K16 acetylation in a dose-dependent manner. Arsenic trioxide downregulated the global histone H4 acetylation at lysine 16 (H4K16ac) via direct binding to histone acetyltransferase human male absent on first (hMOF) in HeLa and HEK293T cell lines. Additionally, HAT in vitro assay showed that arsenic trioxide directly inhibits hMOF activity [273].

#### 4.10.2. Curcumol

Curcumol (Figure 5) is a natural sesquiterpenoid isolated from numerous plants of the Zingiberaceae family and has demonstrated diverse biological properties, such as being anti-inflammatory, anti-proliferative, antioxidant, and antimicrobial [283]. Several research investigations have proven the potent anticancer activity of curcumol; targeting several checkpoints, including HDAC epigenetic mechanism. Curcumol has been shown to suppress choriocarcinoma cancer stem-like cell self-renewal (CSLCs) through HDAC activity (in vitro and in vivo). JEG cells treated with curcumol (75 µg/mL) for sphere formation showed 50% growth inhibition [274]. HDAC enzyme inhibition was assessed using a colorimetric kit assay after seven days of curcumol treatment.

#### 4.10.3. Selenium

Research findings demonstrated that selenium is a potential chemopreventive agent against prostate cancer. In this line, Xiang et al. [275] studied the effect of a single dose of selenite (1.5 µM) for 7 days on the human prostate cancer cell line LNCaP. They reported that selenite could prevent prostate cancer through an epigenetic mechanism by maintaining the expression of GSTP1 promoter, inducing the inhibition of DNA and histone methylation, and enhancing histone acetylation. In addition, selenite treatment altered HDAC expression and significantly affected its activity. It decreased the expression levels of methylated histone H3 on lysine 9 (H3-K9) and increased the expression of acetylated H3-K9 [275].

## 5. Conclusions

In summary, this work has highlighted the close relationship between the enzymes that regulate chromatin architecture and tumor transformation. Indeed, HDACs are responsible, along with other epigenetic modifiers, for maintaining cellular memory during mitotic division. Disturbances of HDAC activities correlate with tumor transformation in several cancers. Through our bibliographic research, we have shown that natural bioactive substances can modify the enzymatic activity of HDAC, and, therefore, its regulation. These remarkable results demonstrate the possible pharmacological effects of these substances and their applications in cancer chemotherapy. However, further testing needs to be performed on these bioactive compounds, to first confirm their safety by investigating their toxicity, and with other in vivo tests, to assess their pharmacokinetics. The validation of these approaches could pave the way for the clinical use of some of these molecules against cancer.

## Figures and Tables

**Table 1:** Natural flavonoids targeting HDAC in human cancers.

Molecules (Origins)	Used Models	Methods	Key Findings	Ref.
(−)-Epigallocatechin-3-gallate (EGCG) (Purchased)	MCF-7 and MDA-MB-231 (Breast cancer cells)	Flow cytometry (apoptosis assay) RT-PCR and real-time PCR (Quantification of *hTERT* expression) DNMTs, HDACs, and HATs activity assays ChIP assay Western blot analysis	Inhibited the transcription of hTERT through epigenetic mechanisms in estrogen receptor (ER)-positive MCF-7 and ER-negative MDA-MB-231 cells. Inhibited the activities of DNMT and histone acetyltransferase (HAT). Remodelled the chromatin structures of the *hTERT* promoter by decreasing the level of acetyl-H3, acetyl-H3K9, and acetyl-H4 in the *hTERT* promoter. Induced chromatin alterations that facilitated the binding of many *hTERT* repressors such as MAD1 and E2F-1 to the *hTERT* regulatory region.	[189]
(−)-Epigallocatechin-3-gallate (EGCG) (Purchased)	A431 (Human skin cancer)	DNA methylation assay HDAC activity assay Western blot analysis Cell lysates	Decreased global DNA methylation levels in A431 cells in a dose-dependent manner. Decreased the HDAC activity. Increased the levels of acetylated lysine 9 and 14 on histone H3 (H3-Lys 9 and 14) and acetylated lysine 5, 12, and 16 on histone H4. Re-expressed the mRNA and proteins of silenced tumor suppressor genes, *p16^INK4a^* and *Cip1/p21.*	[190]
(−)-Epigallocatechin-3-gallate (EGCG) (Purchased)	HT-29 and HCT 116 (Human colon cancer cell lines)	Western blot analysis RNA extraction Real-time PCR	Reduced HDAC and DNMT protein expression. Decreased HDAC2 and HDAC3 expressions. Decreased association between UHRF1 and DNMT3. Decreased association between UHRF1 and HDAC3 in only the HCT 116 cell line.	[187]
(−)-Epigallocatechin-3-gallate (EGCG) (Purchased)	MCF7 and MDA MB 231 (Breast cancer cells)	RT-PCR Western blot analysis	Lowered the protein levels of DNMT1, HDAC1, and MeCP2.	[204]
(−)-Epigallocatechin-3-gallate (EGCG) (Purchased)	HT29 (Human colon cancer cells)	HDAC enzyme activity Western blot	Inhibited the HDAC activity in intact HT29 cells. Decreased the HDAC1 protein level.	[188]
(−)-Epigallocatechin-3-gallate (EGCG) (Not reported)	MCF-7 and MDA-MB-231 (Breast cancer cells)	RNA extraction RT PCR ChIP method Western blot analysis	Reduced levels of the enhancer of zeste homolog 2 (EZH2) and Class I HDAC proteins.	[205]
(−)-Epigallocatechin-3-gallate (EGCG) (Purchased)	CRL-2577, HCT-116 and HT-29HTB-38 cells	Cell viability and apoptosis Real-time quantitative PCR HDAC activity assessment ChIP assay	Combinatorial effects of EGCG and NaB. Increased HDAC1 in RKO CRC cells. Inhibited the HDAC1, DNMT1, and survivin in all the three CRC cells tested. Affected the global DNA methylation.	[206]
(−)-Epigallocatechin-3-gallate (EGCG) (Purchased)	HeLa cells	HDAC activity assessment Bisulfite modification MS-PCR RT-PCR	Decreased the HDAC activity time-dependently. Zn ion was taken to be the substrate-binding site of HDAC1.	[207]
(−)-Epigallocatechin-3-gallate (EGCG) (Purchased)	A549/DDP cell line	Cell viability assay MTT assay RT- PCR Total HDAC activity and DNMT activity in vivo experiments	In vitro EGCG + cisplatin (DDP) treatment caused: Inhibition of DNMT and HDAC activities, reversal of hypermethylated status, and downregulation of the expression of GAS1, TIMP4, ICAM1, and WISP2 gene in A549/DDP cells. In vivo EGCG + DDP pre-treatment caused: Inhibition of tumors, a decrease in methylation levels of GAS1, TIMP4, ICAM, and WISP2, and an increase in their expression levels.	[208]
(−)-Epigallocatechin-3-gallate (EGCG) (Not reported)	APL NB4 and HL-60 cells	RT-qPCR ChIP assay MSP and sequencing Western blot analysis	Downregulated the epigenetic modifiers HDAC1 and HDAC2 Downregulated the polycomb repressive complex 2 (PRC2) core components in gene and protein levels. Reduced gene-binding effect of core components of *EZH2*, *SUZ12*, and *EED.*	[209]
(−)-Epigallocatechin-3-gallate (EGCG) (Not reported)	DUPRO and LNCaP (prostate cells)	RNA extraction RT– semi q-PCR Western blot analysis ChIP assay HDAC enzyme activity	Reduced the expression of both EZH2 enhancers and its catalytic product trimethylation of H3. Increased acetylation of histone H3K9/18. Reduced the activity of Class I HDACs, as well as EZH2 and H3K27me3 levels.	[210]
(−)-Epigallocatechin-3-gallate (EGCG) (Not reported)	HMEC-1 and HUVECs cells	Cell proliferation assay RNA isolation Reverse transcription and RT- PCR Western blot analysis HDAC Activity Assay	Increased histone acetylation (H3K9/14ac, H3ac), as well as methylation of both active (H3K4me3) and repressive (H3K9me3) chromatin. Inhibited HDAC activity in both cellular and cell-free models. Altered epigenome modulator expression and activity (HDAC5 and 7, p300, CREBP, LSD1 or KMT2A).	[191]
(−)-Epigallocatechin-3-gallate (EGCG) (Purchased)	Breast cancer cells	RNA extraction Protein extraction qRT-PCR Western blot analysis ChIP assay HDAC activity HMT (H3K27me3) activity	Induced changes in histone modifications. Resulted in an increased apoptosis.	[211]
Delphinidin (Purchased)	JB6 P+ (Mouse epidermal cells) HepG2–C8 (hepatocellular cancer cells)	Western blot Quantitative real-time PCR Bisulfite genomic sequencing	Reduced DNMTs and HDACs protein expression.	[212]
Apigenin (Purchased)	Human prostate cancer cell lines 22Rv1 and PC-3 (prostate cancer cells)	HDAC activity assessment RNA isolation RT-PCR and q-PCR Western blot analysis ChIP assay Tumor xenograft studies	Inhibited Class I HDACs in prostate cancer cells. Inhibited HDAC enzyme activity, particularly HDAC1 and HDAC3. Induced histone acetylation. Increased p21/waf1 protein and mRNA expression, as well as p21/waf1 mRNA expression. Reduced (in vivo) tumor development, HDAC activity, HDAC1, and HDAC3 protein expression.	[192]
Apigenin (Purchased)	JB6 P+ (Skin cells)	RT-PCR Western blot analysis	Reduced some HDACs (1–8) and their expression levels.	[213]
Apigenin (Purchased)	MDA-MB-231 (breast cancer cells)	Immunoblot analysis HDAC assay Nuclear extract preparation ChIP assay	Inhibited HDAC activity. Induced acetylation of histone H3. Increased the acetylation of histone.	[193]
Galangin (Purchased)	Neuroblastoma cells	Flow cytometry ELISA RT-PCR and qPCR Western blot analysis ChIP assay DNA methylation analysis	Reduced the *BACE1* at mRNA and protein levels. Reduced acetylated H3 in BACE1 promoter areas by increasing endogenous HDAC1-mediated deacetylation.	[194]
Genistein (Purchased)	Human esophageal squamous cell carcinoma cell lines KYSE 510 and KYSE 150 cancer cells	Modification by bisulfite Methylation-specific PCR RT-PCR	Reversed DNA hypermethylation. Reactivated *RARβ*, *p16^INK4a^*, and *MGMT* in KYSE 510 cells. Reversed DNA hypermethylation and reactivated RARβ in KYSE 150 cells and prostate cancer LNCaP and PC3 cells. Activated RARβ. Inhibited the activity of HDAC.	[195]
Genistein (Purchased)	MCF7 and MDA MB 231 (Breast cells)	Bisulfite conversion RT-PCR Western blot	Reduced HDAC1 and MeCP2 protein levels.	[204]
Genistein (Purchased)	A498, ACHN, HEK-293 and HK-2 cells	RT-PCR Sodium bisulfite modification and sequencing HAT and HDAC analysis	Increased HAT activity and reduced HDAC activity.	[205]
Genistein (Purchased)	LNCaP, PC3, and RWPE-1 (Prostate cells)	Quantitative RT-PCR Sodium bisulfite modification and sequencing ChIP analysis HAT and HDAC analysis	Increased the levels of acetylated histones 3, 4, histone three dimethylated at lysine 4, histone 3 trimethylated at lysine 4, and RNA polymerase II. Decreased DNA methyltransferase and methyl-binding domain protein 2 activity. Increased HAT activity.	[196]
Genistein (Purchased)	HT29 (colon cancer cells)	HDAC enzyme activity Western blot analysis	Inhibited HDAC activity in intact HT29 cells. Reduced HDAC1 protein levels.	[188]
Genistein (Purchased)	MCF-7, MDA-MB-231, and MDA-MB-157, HMECs cells, and two mouse models	MTT assay RT-PCR ChIP assay	Reduced the activity of HDAC, alone or in combination with TSA. Reduced binding to the *ERα* promoter, as well as gene expression for HDACs. Reduced HDAC1 protein and mRNA expression in both animal models studied.	[197]
Genistein (Purchased)	Rat colon tissues	RNA isolation qPCR analysis ChIP analysis Bisulfite sequencing	In the post-AOM phase, there was a decrease in H3Ac at the promoter of *Wnt5a*, *Sfrp5*, and *Sfrp2.* Repressed histone H3 lysine 9 methylation and serine 10 phosphorylation at the promoters of *Sfrp2*, *Sfrp5*, and *Wnt5a* in the post-AOM period. In the post-AOM period, the nuclear level of HDAC3 protein was increased. After AOM induction, H3Ac was reduced in the same region of the *Sfrp5* promoter.	[198]
Genistein (Purchased)	MCF-7 and MDA-MB-231 cells	HDAC activity assay HMT activity	Inhibition of HDAC and HMT by GEN + SFN. Downregulation of HDAC2 and HDAC3 levels at the mRNA and protein levels. GEN + SFN downregulated the *hTERT* levels.	[214]
Genistein (Purchased)	Human cervical cancer cells	DNMT and HDAC activity analysis In silico studies in the post-AOM period	Reduced the expression of HDAC and enzymatic activity in a time-dependent manner. Interacted with members of the DNMT and HDAC families. Reversed the tumor suppressor genes’ promoter region methylation, and their expression was restored.	[215]
Genistein (Purchased)	HeLa cells	qPCR HDAC activity assay HMT-H3K9 activity Global DNA methylation	Altered HDACs, HMTs, demethylases, and histone phosphorylases’ expression. Reduced HDAC and HMT activity, as well as global DNA methylation levels.	[203]
Kaempferol (Not reported)	HepG2, Hep3B, and HCT-116 cells	In silico docking analysis HDACi screening assay HDAC inhibition profiling Immunoblotting Real-time cell monitoring	Inhibited the activity of HDAC. Induced hyperacetylation of histone complex H3. Reduced cell viability and proliferation rate.	[201]
Luteolin (Purchased)	MDA-MB231-1833, LNM35, HT29, HepG2, and MCF7/6 cells	HDAC assay and histone acetylation levels	Inhibited HDAC activity.	[216]
Luteolin (Purchased)	HCT116 (Colorectal cells)	HDAC activity assay	Reduced levels of HDAC protein and enzyme activity.	[217]
Pectolinarigenin (Purchased)	143B, HOS, and MG63 (Osteosarcoma)	Western blot analysis RT-PCR ChIP assay MTS cell viability assay	Disrupted the development of the STAT3/DNMT1/HDAC1 complex. Caused an increase in SHP-1 expression in osteosarcoma.	[218]
Pelargonidin (Purchased)	Skin epidermal JB6 (JB6 P+) cells	Western blot analysis RT-PCR Bisulfite genomic sequencing	Reduced protein levels of genes encoding HDACs.	[219]
Silibinin (Not reported)	H1299 cells	HDAC activity RT- RT-PCR assay	Reduced the activity of HDAC in a dose-dependent manner. Reduced HDAC1, HDAC2, and HDAC3 protein levels, whereas HDAC1, HDAC6, SET domain proteins (SETD1A, D4, D6) and lysine-specific demethylases were upregulated (KDM 5B, 5C, 4A).	[199]
Silibinin (Purchased)	SW480 and SW620 (Colon cells)	HDAC activity	No effect on the activity of HDACs.	[220]
Silibinin (Purchased)	DU145 and PC3 (Prostate cells)	Western blot analysis RT-PCR HDACs assay	Reduced HDAC1-2 expression levels in a concentration-dependent manner.	[200]
Taxifolin (Purchased)	HepG2 cells, skin epidermal JB6 P+ cells, and HepG2-C8 cells	Western blot analysis RNA extraction qRT-PCR assay Bisulfite genomic sequencing	Inhibited DNMT and HDAC protein expression.	[221]
Quercetin (Purchased)	HL-60 (leukemia cells)	Western blot analysis RT-PCR HDAC assay HAT assay ChIP assay	Increased histone H3 acetylation, which promoted the production of FasL Activated HAT and inhibited HDAC.	[202]
Quercetin (Purchased)	Hamster buccal pouch (HBP) carcinomas	Immunohistochemistry Western blot analysis RT-PCR	Inhibited HDAC-1 and DNMT1 activities.	[222]
Quercetin (Purchased)	Eca9706 cells	MTT assay Immunoblotting MSP of *p16^INK4a^* gene promoter	Reduced the reverse expressions of global HDAC1. Quercetin + butyrate displayed a reverse effect targeting both altered DNA methylation and histone acetylation, acting as HDAC inhibitor mediated via epigenetic-NF-κB cascade signaling.	[223]
Quercetin (Purchased)	HeLa cells	DNMT and HDAC activity assay HMT-H3K9 activity assay Molecular modeling qRT-PCR	Reduced the activity of HDAC activity. Reduced the activation of HMT-H3K9. Modified the expression of many chromatin modifiers, and lowered the activity of HDACs and HMTs. Several DNMTs and HDACs interacted with residues in their catalytic cavities (as a competitive inhibitor).	[203]

**Table 2:** Natural alkaloids targeting HDAC in human cancers.

Molecules (Origins)	Used Models	Methods	Key Findings	Ref.
Berberine (BBR) (Purchased)	U266, KG1-α, and HL-60 cells	Molecular Docking Growth inhibition assessment RT-PCR Western blot analysis	Affected the enzymes involved in histone acetylation and methylation. Induced cytotoxicity and apoptosis in HL-60/ADR and KG1-α cells. Upregulated histone acetyltransferase CREBBP and EP300, histone deacetylase SIRT3, histone demethylase KDM6A, as well as histone methyltransferase SETD7. Downregulated histone acetyltransferase HDAC8, histone methyltransferase WHSC1I, WHSC1II, and SMYD3.	[224]
Isofistularin-3 (Iso-3) (*Aplysina aerophoba)*	RAJI, U-937, JURKAT, K-562, HL-60, MEG-01, cells	HDAC activity assessment Molecular docking study Western blot analysis	Modified the aryl hydrocarbon receptor (AHR) promoter methylation and increased the AHR expression in RAJI cells.	[225]
Nicotinamide (NA) (Purchased)	Female mice skin	RT-PCR DNA bisulfite modification Western blot analysis	Downregulated the miR-203 levels at 16 weeks. Upregulated the HDAC, DNMT, and promoter methylation of miR-203 at 4 or 16 weeks. Prevented changed gene expression.	[226]
Trichostatin A (TSA) (Purchased)	PANC-1, CAPAN (1 and 2), and KATO-III cells	RT-PCR siRNA Western blot analysis ChIP assay	TSA + 5-aza increased *MUC4* expression in a cell-specific manner. *MUC4* silencing was directly engaged by HDAC3, HDAC1, DNMT3B, and DNMT3A binding to its 5′-UTR in a cell-specific manner. Inhibited the histone deacetylation, associated with strong *MUC4* repression in high-expressing cells	[227]
Psammaplin (*Jaspis* sp. and *Poecillastra wondoensis)*	Ishikawa cancer cells	RT-PCR Western blot analysis	Induced accumulation of acetylated histones and reduced HDAC levels. Upregulated cyclin-dependent kinase inhibitor expression.	[228]
Psammaplin (*Pseudoceratina purpurea)*	A549, MCF7, and W138 cells	HDAC Assay Immunoblotting	Inhibited HDAC1 activity. Exhibited significant cytotoxicity in A549, MCF7, and W138 cells. Increased acetylation of histones.	[229]
Psammaplin (*Pseudoceratina purpurea)*	In vitro enzymatic assays	Cell proliferation assessment HDAC enzyme activity	Inhibited HDAC effectively. Induced weak cytotoxicity.	[230]
TSA (Purchased)	MCF-7, MDA-MB-231, and MDA-MB-157 cells and mouse models	RT-PCR assay Western blot analysis ChIP assay	Reduced the activity of HDAC, either alone or in conjunction with GEN.	[197]
TSA (Purchased)	SKOV3 (Ovarian cells)	Western blot analysis Histone immunoblots assay	TSA + decitabine reduced the DNMT and HDAC activities. TSA + decitabine increased acetylation of histone H3 and H4. TSA + decitabine inhibited the expression of lysine-specific demethylase-1. TSA + decitabine increased the transcription activity marker dimethylated-H3K4, while suppressing dimethylated-H3K9.	[231]

**Table 3:** Natural terpenoids targeting HDAC in human cancers.

Molecules (Origins)	Used Models	Methods	Key Findings	Ref.
Corosolic acid (Purchased)	TRAMP-C1 cells	RT-PCR DNA extraction HDAC activity assessment ChIP assay Western blot analysis	Increased the acetylation of histone H3 lysine 27 (H3K27ac). Reduced trimethylation of histone H3 lysine 27 (H3K27Me3) in the Nrf2 promoter region. Reduced the expression and activity of epigenetically modifying enzymes in TRAMP-C1 cells.	[232]
Cucurbitacin B (*L. graveolense* Roxb.)	NSCLC cells	RT-PCR Western blot analysis HDAC activity Immunohistochemical staining	Altered histone modifications.	[233]
Parthenolide (PTL) (Purchased)	ZR-75-1 and 293T cells	RT-PCR HDAC activity Western blot analysis ChIP assay	Depleted HDAC1 protein, without affecting Class I/II HDAC proteins. HDAC1 depletion and cell death were promoted by the mutant DNA-damage transducer ataxia telangiectasia.	[236]
Parthenolide (PTL) (Purchased)	AML cells	Methylcellulose colony-forming Western blot analysis Flow cytometry	HDAC induced apoptosis was prevented in multiple human AML cells. Increased HDAC-induced lethality. PTL + HDACis caused apoptosis in MLL-ENL cells. PTL + HDACis triggered SAPK/JNK activation.	[237]
Parthenolide (PTL) (Not reported)	JB6 cells	Western blot analysis ChIP method RT-PCR siRNA transfection HDAC activity	Modulated gene expression.	[238]
Parthenolide (PTL) (Purchased)	MDA-MB231 cell line	Cell viability assessment ROS and GSH analysis Western blot analysis	PTL + HDACi induced GSH depletion, Cyt c release, activation of caspase 3 and apoptosis. PTL + HDACi sustained both HDACi-induced hyperacetylation of histones H3 and H4 and PTL-induced downregulation of DNMT1 expression PTL + HDACi boosted PTL’s cytotoxic impact.	[239]
Ursolic acid (Purchased)	JB6 P+ cells	qPCR Western blot analysis HDAC activity assay	Reduced the expression of HDAC1, 2, 3, and 8 (Class I) enzymes and the activity of HDAC6 and 7 (Class II).	[240]
Ursolic acid (Purchased)	Male Sprague-Dawley rats	mRNA expression measurement qPCR Development of PK/PD model	Inhibited the induction of epigenetic markers in leukocytes.	[232]
Z-Ligustilide (LIG) (Purchased)	HS578t, MDA-MB-231 and MDA-MB-453 cells	Western blot analysis Luciferase assay ChIP assay Immunoprecipitation	Increased Ace-H3 (lys9/14) enrichment in the ER promoter. Downregulated the HDAC activity.	[241]

**Table 4:** Natural fatty acids targeting HDAC in human cancers.

Molecules (Origins)	Used Models	Methods	Key Findings	Ref.
Butyrate (Purchased)	Eca9706 cells	MTT assay Immunoblotting Methylation-specific PCR	Inhibited the development of human oesophageal cancer cells. Reduced NF-Bp65, DNMT1, HDAC1, and Cyclin D1 reverse expression. Increased caspase-3 and p16INK4 expression. Butyrate + quercetin acted as HDAC inhibitors through epigenetic-NF-κB cascade signaling.	[223]
Butyrate (Purchased)	CRL-2577, HT-29, and HCT-116 cells	HDAC activity assessment CpG methylation ChIP assay	Inhibited survivin, DNMT1, and HDAC1. Affected chromatin structure and global DNA methylation.	[206]
Butyric acid (Purchased)	Female mice skin	Bisulfite modification of DNA RT-PCR assay	Decreased miR-203 expression. Upregulated the methylation of DNMT, HDAC, and miR-203.	[226]
Eicosapentaenoic acid (EPA) (Purchased)	McRH-7777 cells	Flow cytometry qRT-PCR Immunoblot analysis ChIP method	Reduced HDAC1 and DNMT activity and expression. Promoted tumor suppressor gene expression. Activated PPAR by inhibiting HDAC1 expression in hepatocarcinoma cells. Re-expressed the tumor suppressor gene Hic-1 in response to EPA-bound PPAR.	[243]

**Table 5:** Other natural molecules targeting HDAC in human cancers.

Molecules (Origins)	Used Models	Methods	Key Finding	Ref.
**Isothiocyanate**				
Phenethyl isothiocyanate (PEITC) (Not reported)	HCT116, SW480, and SW620 cells	Cell viability Western blot analysis Quantitative RT-PCR	Induced stable changes in the expression profile of epigenetic erasers/writers. Induced hypomethylation of PcG target genes, which are generally hypermethylated in cancer.	[244]
Phenethyl isothiocyanate (PEITC) (Purchased)	HT29 (Colon cells)	Determination of HDAC1 levels	PEITC + LA reduced the amount of aberrant crypt foci, as well as DNMT1 and HDAC1 levels (in vivo).	[245]
Sulforaphane (SFN) (Not reported)	MCF-7 and MDA-MB-231	RT-PCR Western blot analysis HDAC and HAT activity Bisulfite sequencing analysis ChIP method	Inhibited *hTERT* in both cells. Reduced the trimethyl-H3K9 and trimethyl-H3K27 in active chromatin indicators in a dose-dependent manner.	[189]
Sulforaphane (SFN) (Purchased)	HeLa cells	DNMT and HDAC activity assessment Molecular docking study Bisulfite Modification and MSP RT-PCR	Inhibited the activity of HDAC. Lowered the HDAC1 expression. Reactivated RAR, CDH1, DAPK1, and GSTP1 genes or elevated their expression.	[207]
Sulforaphane (SFN) (Purchased)	A549 and H1299 (Lungs cells)	HDAC activity assay qRT-PCR Tumor xenografts	Inhibited the activity of HDAC in lung cancer cells. Increased histone (H4 and H3) acetylation. Suppressed the activity of HDAC in vivo.	[249]
Sulforaphane (SFN) (Purchased)	MCF-7 and MDA-MB-231	HDACs activity assay	SFN + WA decreased HDAC expression.	[250]
Sulforaphane (SFN) (Purchased)	A549, HEK293, HT29 cells	Bisulfite genome sequencing RT-PCR Western blot analysis ChIP method HDAC and DNMT activity assessment	Reduced CpG methylation in the miR-9-3 promoter region. Increased H3K4me1 enrichment at the miR-9-3 promoter. Induced the expression of miR-9-3. Reduced the epigenetic modifying enzymes’ expression and activity.	[251]
Sulforaphane (SFN) (Purchased)	Breast cancer cells	RT-PCR Western blot analysis HDACs activity Global histone H3 acetylation quantification RNA sequencing analysis	Increased gene transcription in tumor suppressor genes. Decreased the expression of tumor promoting genes. Decreased HDAC1 gene expression and enzymatic activity. Increased levels of histone acetylation.	[252]
Sulforaphane (SFN) (Purchased)	HCT116 and RKO (Colon cells)	RT-PCR Western blot analysis HDAC activity	Reduced the oncogenic *hTERT* mRNA, HDAC, miR-21, protein, and enzyme levels.	[253]
Sulforaphane (SFN) (Purchased)	MCF-7 and MDA-MB-231	RT-PCR Western blot analysis HDAC activity	SFN + GEN suppressed HDAC and HMT. SFN + GEN inhibited the expression of hTERT.	[214]
Sulforaphane (SFN) (Not reported)	HepG2 and GAS cells	RNA-Seq analysis	Inhibited HDAC activity Downregulated chromatin profile regulating enzymes.	[254]
Sulforaphane (SFN) (Purchased)	HepG2 cells	RNA extraction RNA-Seq analysis	Inhibited HDAC activity. Methylation of oncogenic TF binding site motifs affected its activity.	[249]
**Quinones**				
Laccaic acid (LA) (Purchased)	HT29 cells	HDAC activity assay	LA + PEITC reduced HDAC1 expression levels (in vivo).	[255]
Naphthazarin (Naph) (Purchased)	MCF-7 cells	RT-PCR Western blot analysis ChIP method	Activated the p21 promoter by Naph + IR, by inhibiting the binding of multi-domain proteins.	[256]
Thymoquinone (Purchased)	JK cell line and MDA-MB-468 cells	Gene analysis RT-PCR analysis	Downregulated numerous major epigenetic factors.	[257]
**Carotenoid**				
Astaxanthin (Purchased)	LNCaP cells	Cell viability test HDAC activity assay	Inhibited HDAC activity in vitro.	[258]
**Stilbenes**				
Resveratrol (RSV) (Purchased)	MDA MB 231 and MCF7	Bisulfite conversion RT-PCR Western blot analysis	Decreased HDAC1 protein levels.	
Resveratrol (RSV) (Purchased)	HeLa, SiHa, and Caski cervical cells	Promoter methylation analysis Transient silencing of *UHRF1*	Induced PAX1 reactivation via its effect on HDAC, which is mediated through the downregulation of UHRF1, which may affect both histone acetylation and DNA methylation.	[259]
Resveratrol (RSV) (Not reported)	B16F10 and Tumor-bearing mouse model	Western blot analysis Luciferase reporter assays Bisulfite sequencing PCR ChIP assay	HDAC1 was recruited to the focal adhesion kinase (FAK) promoter region.	[260]
Resveratrol (RSV) (Purchased)	MDA-MB-231 and MCF-7 cells	MTT assay Clonogenic method HDAC activity	Reduced HDAC activity.	[261]
Curcumin (Cur) (Purchased)	Hep3B cells	Histone purification Histone acetylation assay Western blot analysis HAT assay HDAC assay RT-PCR	Inhibited histone acetylation in the presence or absence of TSA. Inhibited the activity of HAT both in vitro and in vivo. Induced histone hypoacetylation in vivo.	[262]
Curcumin (Cur) (Purchased)	Raji cells	Immunocytochemistry analysis Western blot analysis	Reduced HDAC1, HDAC3, and HDAC8 protein expression levels. Increased Ac-histone H4 protein expression.	[263]
Curcumin (Cur) (Purchased)	Raji cells	MTT assay Western blot analysis RT-PCR	Decreased the amounts of HDAC3, HDAC1, and p300. Reversed the protection degradation of p300 and HDAC1 by MG-132.	[264]
Curcumin (Cur) (Purchased)	Hela cells	HDAC activity assessment	Inhibited the HDAC activity (IC_50_ = 115 μM).	[265]
Curcumin (Cur) (Purchased)	DAOY, D283 Med, and D341 Med (medulloblastoma)	HDAC activity In vivo medulloblastoma xenografts	Reduced the HDAC4 activity and expression. Increased tubulin acetylation.	[266]
Curcumin (Cur) (Purchased)	LNCaP cells	Western blot analysis DNA extraction and bisulfite genomic sequencing PCR array RNA isolation and RT-PCR ChIP assay Histone H3 methylated lys27 ELISA HDAC activity assay	Altered HDAC expression. Increased the expression of HDAC1, 4, 5, and 8, but decreased the HDAC3. Decreased HDAC activity. Decreased H3K27me3 enrichment at the Neurog1 promoter region and at the global level.	[267]
Curcumin (Cur) (Purchased)	MCF7 and MDA MB 231	Bisulfite conversion and MSP RT-PCR Western blot analysis	Decreased protein levels of HDAC1.	
Curcumin (Cur) (Purchased)	HeLa, SiHa, and Caski cells	MTT assay Quantitative expression of *PAX1*, *UHRF1*, and *p21* *PAX1* promoter methylation analysis Transient silencing of *UHRF1*	Induced reactivation of *PAX1,* due to its effect on HDAC.	[259]
Calebin-A (Not reported)	MPNST cells	Cell proliferation Western blot analysis Flow cytometry ChIP assay	Reduced the expression of acetyl H3 proteins.	[268]
Pterostilbene (PTS) (Purchased)	MDA-MB-157, HCC1806, and MCF10A	RT-PCR Western blot analysis SIRT activity assay DNMT activity	RSV + PTS downregulated the SIRT1 (a type III HDAC).	[269]
**Steroids**				
Guggulsterone (Purchased)	Breast cancer cells	Bisulfite conversion and MSP RT-PCR Western blot analysis	Decreased protein levels of HDAC1.	
Withaferin A (WA) (Purchased)	Breast cancer cells	Bisulfite conversion and MSP RT-PCR Western blot analysis	Decreased HDAC1 and MeCP2 protein levels.	
Withaferin A (WA) (Purchased)	Breast cancer cells	qRT-PCR Western blot analysis HDAC activity	WA + SFN decreased the HDAC activity and expression.	[250]
**Phenolic acids**				
Chlorogenic acid (Purchased)	Breast cancer cells	In vitro HDAC inhibition	Inhibited the HDAC activity (IC_50_ = 375 μM).	[265]
Caffeic acid (Purchased)	Breast cancer cells	In vitro HDAC inhibition	Moderately inhibited the HDAC activity (IC_50_ = 2.54 mM).	[265]
**Secoiridoids**				
Oleacein (*Olea europaea* L.)	U266, NCI-H929, RPMI-8226, MM1s, and JJN3 cells	Western blot analysis qRT-PCR HDAC activity	Accumulated α-tubulin and acetylated histones. Downregulated multiple Class I/II HDACs. Inhibited HDACs with downregulation of Sp1.	[270]
**Tannins**				
Ellagic acid (EA) (Purchased)	hASCs cells	qPCR Western blot analysis HDAC activity Immunocytochemistry of H3R17me2 and HDAC9	Inhibited HDAC 9 downregulation. Reduced the histone acetylation levels.	[271]
**Tanshinone**				
Tanshinone IIA (Purchased)	HepG2-C8, JB6 P+, JB6-shNrf2 cells	Luciferase reporter activity assay RNA extraction and qRT-PCR Western blot analysis HDAC activity Bisulfite sequencing ChIP method	Inhibited HDAC activity.	[272]
**Others natural compounds**				
Arsenic trioxide (AS_2_O_3_) (Purchased)	HeLa or HEK293T cells	Luciferase reporter assay RT-PCR ChIP method Flow cytometry	Reduced global histone through direct binding. Increased HDAC4 expression. Inhibited the hMOF activity.	[273]
Calcium glucarate (CAG) (Purchased)	Female mice cells	RT-PCR Bisulfite sequencing assay Western blot analysis	Downregulated the levels of miR-203 Upregulated DNMT, HDAC, and promoter methylation of miR-203.	[226]
Proanthocyanidins (GSPs)	Breast cancer cells	Western blot analysis HDAC activity	Reduced HDAC activity.	[261]
Curcumol (Not reported)	CSLCs cells	qRT-PCR Western blot analysis Global DNA methylation HDAC activity Xenograft tumorigenicity assay Immunohistochemistry	Blocked DNMT/HDAC activity and CSLC self-renewal in vivo and in vitro. Affected CLSCs by HDAC regulation.	[274]
Selenium (Se) (Not reported)	LNCaP cells	HDAC activity	Reduced HDAC activity. No alteration of mRNA and protein levels of HDACs. Reduced methylated histone H3 on lysine 9 (H3-K9) levels. Increased acetylated H3-K9 levels.	[275]

## Data Availability

No applicable.
